# Tooth Regeneration: Insights from Tooth Development and Spatial-Temporal Control of Bioactive Drug Release

**DOI:** 10.1007/s12015-019-09940-0

**Published:** 2019-12-13

**Authors:** Delan Huang, Jianhan Ren, Runze Li, Chenyu Guan, Zhicai Feng, Baicheng Bao, Weicai Wang, Chen Zhou

**Affiliations:** Guanghua School of Stomatology, Hospital of Stomatology, and Guangdong Provincial Key Laboratory of Stomatology, Sun Yat-sen University, Guangzhou, China

**Keywords:** Tooth development, Spatial-temporal control of drug release, Cytokines, Biodegradable materials, Tooth regeneration

## Abstract

Tooth defect and tooth loss are common clinical diseases in stomatology. Compared with the traditional oral restoration treatment, tooth regeneration has unique advantages and is currently the focus of oral biomedical research. It is known that dozens of cytokines/growth factors and other bioactive factors are expressed in a spatial-temporal pattern during tooth development. On the other hand, the technology for spatial-temporal control of drug release has been intensively studied and well developed recently, making control release of these bioactive factors mimicking spatial-temporal pattern more feasible than ever for the purpose of tooth regeneration. This article reviews the research progress on the tooth development and discusses the future of tooth regeneration in the context of spatial-temporal release of developmental factors.

Several cytokines/growth factors are involved in the precise and directional development of specific tissues and organs. In the craniomaxillofacial region, the development of teeth depends largely on the orderly interaction between the ectodermal epithelium and the mesenchyme [1].

The tooth development process is generally divided into the initiation stage, the bud stage, the cap stage and the bell stage (Fig. 1). At the initiation stage, the epithelial tissue known as the dental placode, locally thickens, and continues to develop into the tooth bud [2]. Meanwhile, the mesenchymal tissue near the tooth bud, aggregates to form the tooth germ. Through the proliferation and folding of the epithelial tissue, the buds gradually evolve to the cap and bell stages. Clusters of undifferentiated epithelial cells, known as the enamel knot, can be observed at the center of the inner enamel epithelium. Each tooth germ has only one primary enamel knot. When the primary enamel knot disappears, secondary enamel knots will appear at the prospective apex of the molars. The enamel knot is considered to be the signal center that controls the shape of the cusp [3]. Subsequently, the epithelial tissue forms odontoblasts and ameloblasts, that lead to the formation of the dentin and the enamel, respectively. After the crown formation, the cervical loop of the dental epithelial cells, continues to elongate and forms a double-layered epithelial structure, found between the dental follicle and the dental papilla, and named the Hertwig’s epithelial root sheath (HERS). Conventionally, researchers believe that HERS is the signal center of the root formation [4].

**Fig. 1:**
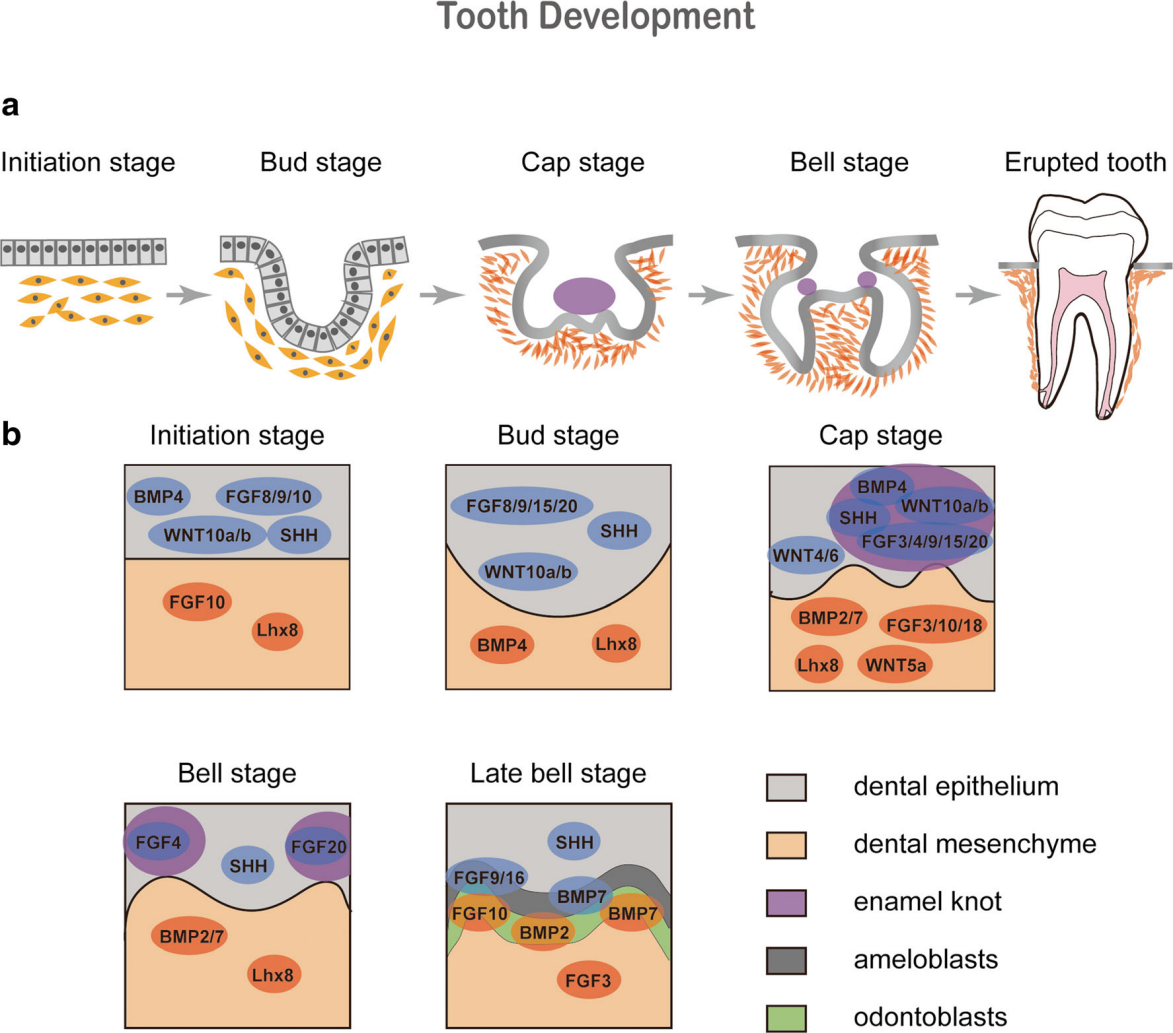
**Spatial-temporal expression of developmental signal molecules during tooth development.** Tooth morphogenesis is divided into the initiation, bud, cap and bell stages. Expression of the fundamental signal molecules in the epithelium and mesenchyme are shown and corresponding to each stage

Many studies have shown that cytokines/growth factors such as BMPs, FGFs, SHHs, WNTs and TNFs, play an important role during this process [1]. Moreover, the expression of these cytokines is characterized by a spatial-temporal specificity [5–7] (Fig. 1). Aberrant expression may lead to tooth development abnormalities [1]. The spatio-temporal control of the developmental cues might be the future for tooth regeneration applications.

With advances in developmental biology and drug delivery, tooth regeneration would be more promising than ever before (Fig. 2). In the following sections, we summarize recent advances in developmental biology and discuss the clues for tooth regeneration in the context of the spatial-temporal control of bioactive drug release.

**Fig. 2:**
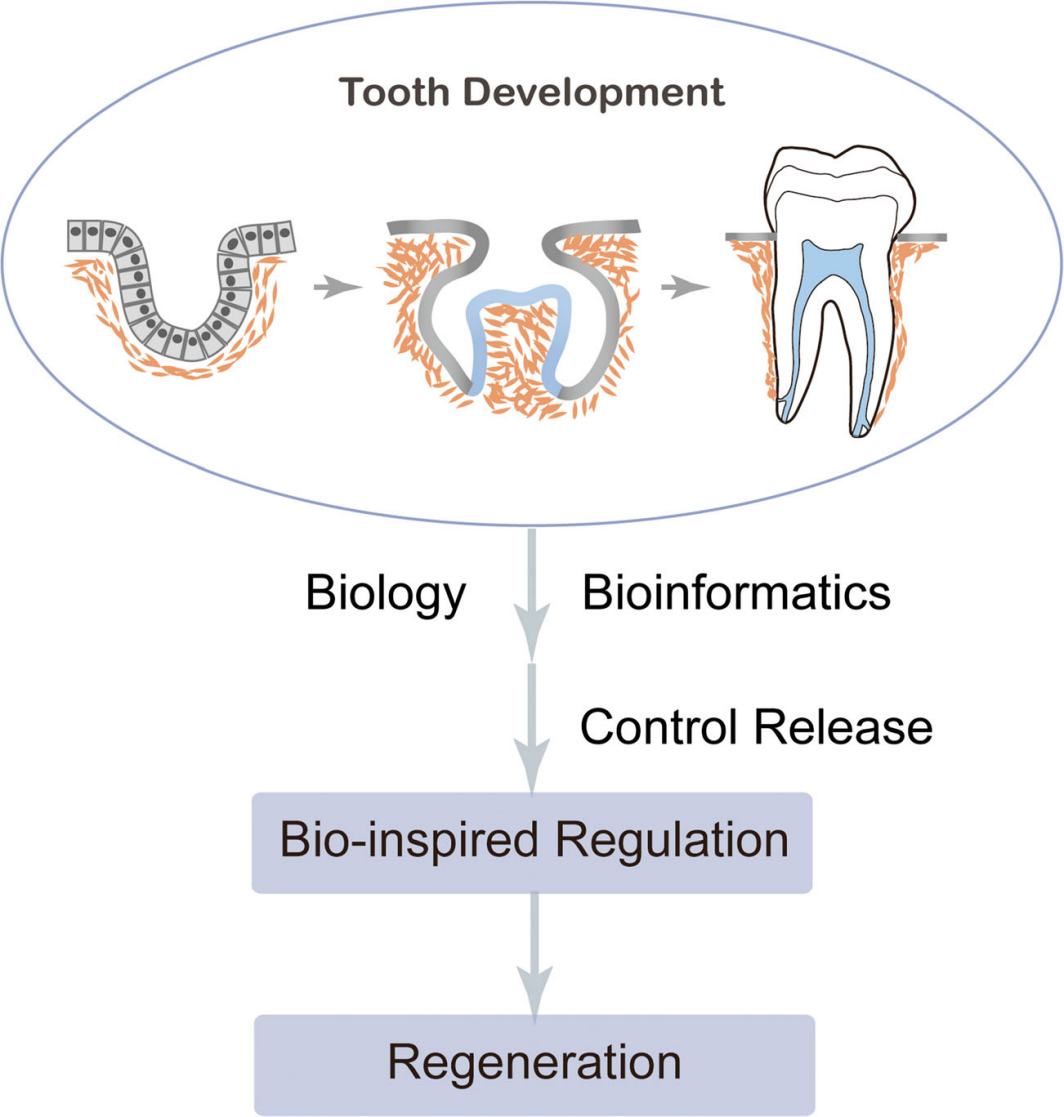
**Schematic representation of the bio-inspired dental regeneration strategy.** The gene expression pattern during tooth development is obtained by biology and bioinformatics, and the development associated with spatial-temporal specific expression could be approached by using different control release strategies for regeneration purpose, and making the goal of tooth regeneration expectable

## Cytokines/ Growth Factors and Tooth Development

BMP, FGF, WNT and SHH signaling pathways are known signaling pathways in tooth development (Tables 1 and 2). Recently, other signaling pathways, such as TNF [8], YAP-Hippo [9] and mTORC1 [10], have also been found to be involved in the process.

**Table 1:** Expression profile of the developmental cues involved in tooth development

Expression stages	Signal molecules	Expression sites
Initiation stage	BMP4, FGF8, FGF9, WNT7b, SHH	Dental epithelium
	FGF10	Dental epithelium Dental mesenchyme
Early bud stage	FGF8, FGF9, FGF15, FGF20	Dental epithelium
	BMP4	Dental mesenchyme
Late bud stage	BMP4, FGF3, FGF4, FGF9, FGF15, FGF20	Primary enamel knot
	FGF3, FGF10, FGF18	Dental mesenchyme
	FGF16, FGF17	Cervical loop mesenchyme
Initiation and bud stages	WNT10a, WNT10b	Dental epithelium
Bud and cap stages	SHH	Enamel knot
Cap stage	BMP2, BMP7	Dental epithelium Dental mesenchyme
	WNT10a, WNT10b	Enamel knot
	WNT4, WNT6	Dental epithelium
	WNT5a	Dental mesenchyme
Early bell stage	BMP2, BMP7	Dental papilla
Bell stage	FGF4, FGF20	Secondary enamel knots
	FGF9, FGF16	Ameloblasts
	SHH	Inner enamel epithelium Stratum intermedium cells
Late bell stage	BMP2	Odontoblasts
	BMP7	Odontoblasts Ameloblasts
	FGF3	Dental papilla
	FGF10	Odontoblasts

**Table 2:** Functions of key cytokines during tooth development

Signaling pathways	Key cytokines	Functions	References
BMP	BMP2	Promotes early tooth mineralization	(Malik et al., 2018)
	BMP4	Coincides with the odontogenic potential; regulate the formation of the Hertwig’s epithelial root sheath	(Jia et al., 2016) (Hosoya, Kim, Cho, & Jung, 2008)
	BMP7	Promotes early tooth mineralization	(Gao et al., 2018)
	BMP9	Promotes odontoblastic differentiation and osteogenic differentiation	(Huang et al., 2019)
FGF	FGF8	Epithelial cell-originating factor	(Trumpp, Depew, Rubenstein, Bishop, & Martin, 1999)
	FGF9	Plays an important role in epithelial invagination and initiates ectodermal organogenesis	(Tai, Chen, Lin, Ling, & Chen, 2012)
WNT	WNT7b	Positions the sites of tooth formation	(L. Sarkar et al., 2000)
	WNT3a	Promotes cementoblast differentiation	(Nemoto et al., 2016)
SHH	SHH	Stimulates epithelial cell proliferation	(Cobourne, Hardcastle, & Sharpe, 2001)

### BMPs

BMPs are a group of multifunctional homologous dimer proteins, which are members of the TGF-β superfamily. Several studies suggest that BMPs are involved at the start and during tooth development. Among BMP family members, BMP4 is a key Msx1-dependent mesenchymal odontogenic signal, that participates in the process of tooth morphogenesis through the bud-to-cap transition [11]. BMP4 expression begins in the dental lamina epithelium, and in the mesenchyme during tooth bud formation, indicating that the odontogenic potential, originally in the epithelium, is transferred to the mesenchyme [12]. Then, by the late bud stage, its expression is found in the primary knot of the dental epithelium [13]. Mechanistically, inhibitors of tooth development such as Dkk2 and Osr2 expressed in the tooth mesenchyme are suppressed by BMP4 signaling, while Msx1 synergizes with BMP4 in activating mesenchymal odontogenic potential that is essential for tooth morphogenesis [11].

Other BMP members that closely relate to tooth development include BMP2 and BMP7. A recent study in miniature pigs showed that BMP2 and BMP7 are expressed in the epithelium and the mesenchyme during the cap stage. At early bell stage their expression decrease and are mainly present in the dental papilla [14]. During the late bell stage, the expression of BMP2 is mostly found in odontoblasts, which implies that it may participate in early tooth morphogenesis and in late odontoblast differentiation and mineral secretion [14]. Another recent study showed that BMP2 played an early temporal, non-redundant role in organic tooth mineralization [15]. BMP7 expression is similar to that of BMP2, but is also detected in ameloblasts [14]. Recently, Huang et al. found that BMP9 regulates tooth development by promoting odontoblastic differentiation and osteogenic differentiation, which was unknown. The BMP9 knockout mice displayed abraded incisor tips, smaller molar cusps and shorter molar roots [16].

### FGFs

FGFs are widely expressed in invertebrates and vertebrates. They are secretory protein ligands that maintain their functions in development, tissue homeostasis and metabolism in autocrine, endocrine or paracrine manners. Several members of the FGF family are involved in odontogenesis [17]. During the initiation stage, the expression of FGF8 and FGF9 are detected in the prospective tooth region of the dental epithelium [17], suggesting that they may take part in the initiation of tooth development. Meanwhile, FGF10 is detected in the dental epithelium and the dental mesenchyme [18]. Unlike FGF8, FGF9, FGF15 and FGF20 that are expressed in the epithelium following the formation of the dental lamina, FGF10 expression is decreased [18]. The expression of FGF3,FGF4, FGF9, FGF15 and FGF20 are detected in the primary enamel knot after its formation; while, FGF3, FGF10, and FGF18 are found in the mesenchyme [19]. The expression of FGF16 and FGF17 are detected in the mesenchyme of cervical loop [19]. At the bell stage, FGF4 and FGF20 expression are restricted to the forming cusps of the secondary enamel knots; while, the expression of FGF9 and FGF16 are detected in the differentiating ameloblasts [17; 19]. The expression of FGF3 is found in the dental papilla at late bell stage; while, FGF10 is expressed in the differentiating odontoblasts [18].

A previous study has shown that FGF8 can induce the expression of Pax9 in mice, revealing the prospective odontogenesis locations, and its essential role reaching beyond the bud stage of tooth development [20]. Another recent study showed that in the first branchial arch (BA1), and using ectoderm Nestin-Cre, that conditional FGF8 knockout leads to the decrease in Pax9 expression in the expected molar region, and the inhibition of molar formation [21]. Moreover, FGF8 expression in the oral epithelium determines the rostral–caudal polarity in BA1 by inducing Lhx8 expression in neural crest–derived mesenchyme, which makes Lhx8 intensively and exclusively expressed in neural crest derived ectomesenchyme and dental mesenchyme. This expression continues to be restricted to the dental papilla and the odontoblast, and gradually decreases over time [22]. The differentiation and function of the dental mesenchyme are regulated by Lhx8 via WNT and TGFβ pathways [23]. FGF9 is also involved in epithelial invagination and initiation of ectodermal organogenesis [24].

### WNTs

The WNT family consists of a group of secretory glycoproteins that are rich in a conserved cysteine sequence, and that regulate cell growth, development, migration and differentiation during embryonic development. The WNT signaling pathway can be separated into the canonical signaling pathway, namely WNT/β-catenin pathway, and the noncanonical pathway, that includes the planar cell polarity pathway and the WNT/Ca^2+^ pathway. It plays critical roles in the initial stage of tooth development, with most of the signaling molecules being specifically expressed in the dental epithelium [25].

WNT7b is expressed in the oral epithelium but not in the presumptive dental epithelium, when tooth forming sites and tooth patterning are defined [26]. WNT7b seems to interact with SHH signaling to delimit boundaries between the oral and the dental ectoderm, and which determine the sites of tooth formation [26]. When the dental epithelium thickens, WNT10a and WNT10b are found expressed in the dental epithelium and these expression remain during the bud stage [27]. At the cap stage, the expression of both genes can be detected in the enamel knot. Meanwhile, WNT4, WNT6 and one of the WNT receptor MFz-6 are specifically expressed in the dental epithelium; while, WNT5a, sFrp2, and sFrp3 are expressed in the dental mesenchyme [28]. A recent study in miniature pig showed that the cusp patterning and the crown calcification may depend on the spatial-temporal distribution of WNT signaling [29]. A mutation in Lef1 gene, a critical component of WNT signaling pathway, caused tooth loss [30]. Odontoblast-specific deletion of the Wls gene, a chaperone protein that regulates WNT sorting and secretion, leads to the inhibition of odontoblast maturation and root elongation via reducing the activity of the canonical WNT signaling [31].

At the initial stages of tooth development, many signaling pathways function downstream of WNT/β-catenin signaling. When the WNT/β-catenin pathway is overactivated, the epithelial markers sonic hedgehog (SHH), Epiprofin (Epfn) and FGF8 are upregulated and ectopically expressed [32]. Mutations in WNT10b, WNT10a, LRP6 and other genes involved in this pathway showed tooth agenesis with or without other ectodermal anomalies [33].

### SHHs

The mammalian hedgehog (Hh) family includes the sonic hedgehog (SHH), the Indian hedgehog (IHH) and the desert hedgehog (DHH) pathways, that encode SHH, IHH and DHH proteins, respectively. Among the three members, SHH is the only Hh ligand that is expressed in teeth [34]. The expression of SHH is present in the oral epithelium prior invagination, and in the tooth epithelium during the tooth development [1]. SHH expression, that begins at the bud stage, is restricted to the enamel knot at the cap stage [35]. It is also expressed in the surrounding inner enamel epithelium and in the stratum intermedium cells during the following stages [36]. The decrease or loss of SHH expression leads to a cap stage tooth rudiment, which has a severely disrupted morphology [37]. SHH also plays vital roles in the development of periodontal tissue [38]. As described above, BMP, WNT and SHH signals are interconnected during tooth development. The differential fate of epithelial stem cells, in mouse molars and incisors, is defined by BMP/SHH signaling network [39]. When reducing SHH function in the epithelium, WNT and FGF signaling are upregulated [40].

### Other Factors

The EDA (ectodysplasin A)-EDAR (ectodysplasin A receptor) system has also been found to be involved in tooth development. It regulates interactions within or between epithelial and mesenchymal cells, and tissues functions by controlling NF-κB-mediated transcription of effectors or inhibitors of the WNT, SHH, FGF and TGF-β pathways [41]. Mutation in Tabby and identified as Ectodysplasin A1 (EDAA1), displays a characterized tooth phenotype, associated with significant reduction in the size and number of molar cusps, and frequent absence of incisors and third molar in the studied mice [42]. Another recent study suggested that EDA mutations cause non-syndromic tooth agenesis [43].

## Dental Regeneration Via Reactivating the Developmental Cues

**Fig. 3:**
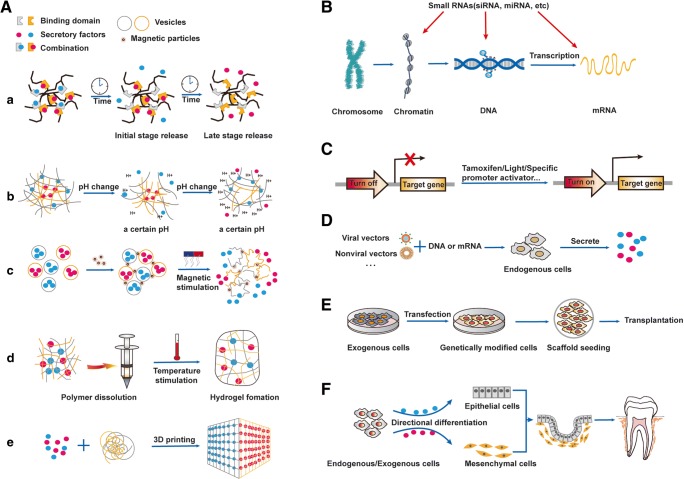
**Strategies for tooth regeneration by reactivating developmental cues. A** Different control release strategies of secretory factors based on biological materials. a) Self-degradation; b) pH-responsive release; c) Magnetic release; d) Thermal release; e) 3D printing. **B** Small RNAs are involved in different parts of the gene expression process. **C** Different turn-on/off systems for spatial-temporal control of gene expression. **D** In vivo delivery of gene expression system. **E** Transplantation of genetically modified cells. **F**Under the above strategies, cells from different sources can be directed to differentiate into specific cells and eventually achieve tooth regeneration

Dental regeneration medicine represents an attractive multidisciplinary approach that offsets traditional dental restoration techniques. As mentioned above, a variety of cytokines participate in different stages of tooth development and in a spatial-temporal manner [1]. The control release of the cytokines for dental regeneration is appealing and is being implemented. Its development depends on research progress in biomaterials, stem cell biology and in other scientific technologies (Fig. 3).

### Control Release of Secretory Factors

#### Biomaterial Based Control of Secretory Factors Releases

Self-degradation is based on the rate of materials degradation in a specific physiological environment, to achieve the spatial-temporal sustained release of cytokines (Fig. 3A a). Although this technique has been widely used in tissue engineering scaffolds, traditional techniques have significant drawbacks, such as high initial release and low bioactive molecular activity. In order to inhibited the burst release of cytokines and enhanced structural stability, many scholars are committed to inventing various kinds of better materials. Fahmy and his co-workers used a low dose of rBMP2 loaded on a resorbable bioactive ceramic to accelerated bone regeneration [44]. Recently, chirality-controlled enzyme-responsive protein nanocapsules were shown to alter the degradation rate by changing the constituent ratio of the material composition, resulting in enhancing wound healing and tissue repair in vivo via the delivery of multiple proteins in a spatiotemporal manner [45]. Affinity interaction is an alternative strategy to achieve sustained release of cytokines. In tissue engineering, the most common way to improve the release kinetics is through heparin-immobilized scaffolds that immobilize cytokines [46]. Wu et al. showed that heparin-based coacervate of FGF2 played a synergistic role with cell proliferation and endogenous facilitated VEGF in improving skin wound healing [47]. In addition, assembly technology like layer-by-layer self-assembly [48] and electrospinning [49] have also made it possible to sustain the release of bioactive molecules in a spatial-temporal manner.

#### The pH-Responsive Release System

The pH release system regulates cytokines release rate by stimulating the response through pH changes (Fig. 3A b). In a recent review, the assembly structure and slow-release behavior of pH-responsive polymers were analyzed and the potential applications of this kind of materials were prospected [50]. pH-responsive release systems have special significance in tissue regeneration due to pH variations in human tissues and organs, which has begun to be applied to diagnosis [51] and treatment of some diseases [52]. Some pH sustained-release materials have been invented, but there is still a lack of in vivo experiments to prove their application in regenerative medicine [53, 54].

#### Magnetic and Thermal Release Technology

Nanotechnology, nanocapsules, liposomes, vesicles and other particle/nanoparticle carrier systems are widely investigated for efficient delivery of growth factors. Magneto-nano technology is one of them and its targeting ability can be divided into two different classes [55]. The first class includes magnetic fields to magnetize carriers and magnetic gradients for targeting and that are provided by external magnets. The second class relies on the combination of magnets implanted into the target area and external magnets. Controlled cytokines release can be achieved by directly and thermally heating responsive polymer particles containing magnetic nanoparticles and cytokines, or by secondary heating, where the thermally responsive particles containing cytokines are surrounded by heated magnetic particles. The local temperature increase leads to cytokines release from thermally responsive polymer particles (Fig. 3A c-d) [55]. Magnetic field and magnetic response scaffolds had been used to improve bone repair and regeneration [56]. Fan M et al. have developed nanometers of gelatin chitosan and heparin based on magnetic biopolymers for BMP-2 [57]. In another study, SAOS-2 cells cultured on the gel, combined with bFGF and human serum albumin coated Fe3O4 nanoparticles, have noticeably enhanced alkaline phosphatase activity and calcium deposition activity [58].

#### 3D Printing Technology

The 3D printing technology is a rapid proto-typing and additive manufacturing technology, which manufactures complex architecture via a layer-by-layer building process and with high precision (Fig. 3A e) [59]. The flexibility and controllability of 3D bioprinting enable complex and customized release profiles of multiple cytokines to achieve spatial-temporal gradients that regulate cellular functions in tissue or organ regeneration [60, 61]. Moreover, many studies have promoted the application of 3D printing technology in cytokine sustained-release by improving processing [62], advancing technology [63] or allowing combinations with other forms of carriers [64]. Up to now, these materials have been successfully used in various tissue and organ regeneration experiments in vitro and in vivo, such as vascular regeneration [65], bone regeneration [63] and skin regeneration [66]. The 4D printing technology is a dynamic and time dependent manufacturing process based on advanced 3D-print features, which providing great potential for tissue and organ engineering applications [67].

### Control Delivery of Small RNAs

Small RNAs including small interfering RNAs (siRNAs) and microRNAs (miRNAs), are part of the short chain RNAs in non-coding RNAs (ncRNAs) (Fig. 3B). SiRNAs are double-stranded RNAs that downregulate gene expression guided by sequence complementarity with the target mRNA. Since its first discovery in 1998 [68], its delivery strategy has developed rapidly. So far, many different siRNA delivery approaches including siRNA conjugates and lipid nanoparticles, have been applied to disease treatment and tissue regeneration [69]. For example, Zhang et al. developed a targeting system for delivering siRNAs to markedly promoted bone formation [70]. More recently, Castleberry et al. developed an ultrathin polymer coating to sustain the local delivery of siRNA so as to improve wound healing in diabetic mice [71]. Furthermore, the potential toxicities of these technology have been gradually discovered. These include but not limited to on-target effects, sequence-specific off-target effects, immune activation and toxicity associated with the delivery vehicles [72].

MiRNAs can simultaneously identify hundreds of target mRNAs with multiple miRNAs working together for the same mRNA [73]. A As post-transcriptional gene regulators, they can target and disassemble mRNAs or repress their translation [74]. Many studies have shown that miRNAs play a significant regulatory role in tissue repair and regeneration, such as wound healing [75], cardiac repair [76]. In vivo delivery of exogenous miRNAs provides an effective way to regulate gene expression during tissue repair and regeneration, which was proved and validated in mice [77] and zebrafishs [78]. To optimize miRNA delivery, Zhang et al. developed a cell-free 3D scaffold with biodegradable microspheres, that spatially regulated the release of miR-26a to repair critically-sized bone defects in osteoporotic mice [79]. Zhou et al. used miR-126-loaded electrospun membranes for miRNAs local delivery to improve blood vessel regeneration [80]. Moreover, a recent study showed that intracardiac injection of a single administration of synthetic miRNA-lipid formulations enhanced cardiac repair in mice after myocardial infarction [81].

### Spatial-Temporal Delivery of Gene Expression Systems

Delivery of gene expression systems that produce locally nascent proteins in vivo, is more advantageous compared to traditional methods for products delivery. In recent years, research on genes-controlled expression has rapidly developed. Some important and potential technologies will briefly be introduced below, and their combinations will also be discussed (Figure C-D).

#### Spatial-Temporal Control of Gene Expression

##### Hormone Induction

All kinds of hormones participate in development and regeneration stages. Steroid hormones function by binding to receptor proteins in the cytoplasm of target cells to form hormone-receptor complexes, which enter the nucleus and bind to specific chromosomal sites to regulate the transcription of specific genes. For example, estrogens play pivotal roles in various physiological processes, most of which are mediated by the estrogen receptors alpha (ERα), beta (ERβ) and G protein-coupled receptor 30 (GPR30). Many studies have used estrogen-inducible promoters to modify gene expression systems to regular gene expression [82–84]. Senturk et al. optimized a CRISPR/Cas9 system by combining it with an FKBP12-derived destabilizing domain and an inducible Cre-estrogen receptor fusion domain, which enabled rapid and tunable gene editing [85].

##### Optogenetics Regulation

Optogenetics is a rapidly developing bioengineering technology which integrates many subjects, such as optics, software control technology, genetic engineering technology, electrophysiological technology. It was originally applied in the field of neurology and a recent review indicated that it could control nerve growth and neurotrophic factor expression in a precise spatial and temporal manner [86]. The light-based mechanisms can activate or inhibit the expression of target genes in the FGF [87], WNT/β-catenin [88] and TGF-β signaling pathways [89] by light-induced conformational change of various photoactivatable proteins or photocaging/uncaging of effectors [90]. Yang et al. created the LightON system, a light-switchable transgene system, which can initiate spatiotemporal expressions of target transgenes in mammalian cells, upon light stimulation [91]. However, potential toxicity associated with the high expression was reported by a study of zebrafish embryogenesis, which may limited its application [92]. To overcome this obstacle, the blue-light activated EL222 system, renamed TAEL was invented, and which drived the expression with minimal toxicity [93, 94]. In addition, some studies have used optical gene elements to link Cre recombinase to regulate DNA recombination [95, 96]. Recently, Nguyen et al. combined genetically encoded photo-switchable calcium actuators with dCas9 to control gene expression, overcoming some limitations of the CRISPR/Cas9 (dCas9) system [97]. Simultaneously, a CRISPR-dCas9 effector device that is activated by far-red light (FRL), engineered by Shao and his research team, efficiently promoted the differentiation of induced pluripotent stem cells (iPSCs) into functional neurons by up-regulating NEUROG2, a single neural transcription factor [98].

##### Dental Development-Related Specific Promoters

In the process of tooth development, some site-specific promoters like WNT1 promoter, play a vital role in regulating the orderly expression of genes. WNT1 encodes the signaling protein WNT1, involved in the canonical WNT pathway. Previous research has shown that the expression of WNT1 is restricted to the migrating neural crest cells, which contribute to tooth and mandible development [99]. Simultaneously, Chai et al. successfully constructed a transgenic model under the control of the WNT1 promoter [99]. Up to now, this conditional knockout model of transgenic mice has been widely used in the study of tooth development and regeneration [100–102].

In addition, dentin matrix protein 1 (DMP1) produced by odontoblasts and osteoblasts is mainly expressed in bone and dentin [103]. Jacob et al. showed that TCF11, which could specifically bind to the DMP1 promoter, played a significant role in regulating the transcription of DMP1 in odontoblasts and osteoblasts [103]. This provides a way to spatiotemporally regulate the expression of DMP1.

#### In Vivo Delivery of Gene Expression System

**Fig. 4:**
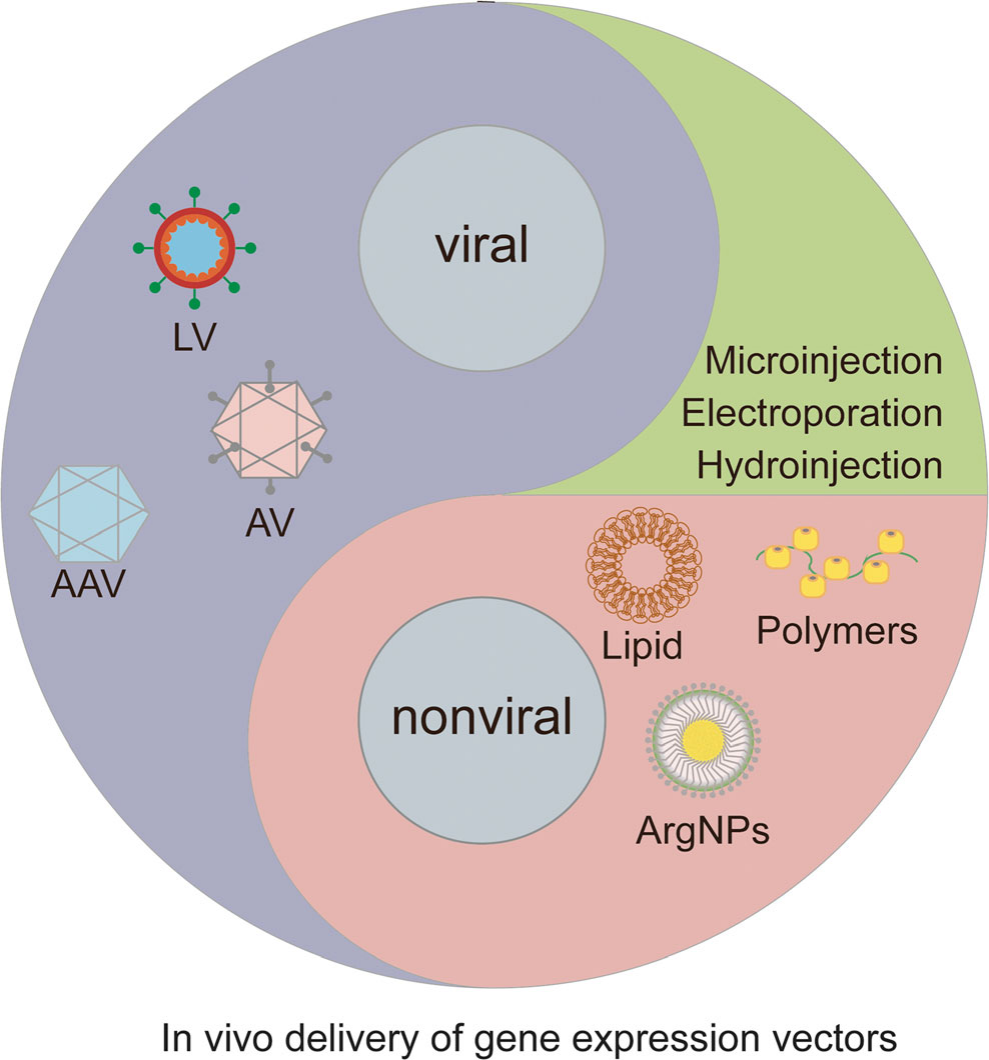
**In vivo gene delivery strategies.** The gene expression systems could be delivered non-virally and virally, both of which have advantages and disadvantages. LV, lentivirus; AV, adenovirus; AAV, adeno-associated virus; Lipid, liposome; ArgNPs, cationic arginine gold nanoparticles

The in vivo gene delivery strategy can be generally divided into viral and non-viral vector delivery systems (Fig. 4). Viral vectors including oncoretroviruses, lentiviruses (LVs), adenoviruses (AVs) and adeno-associated viruses (AAVs), have relatively high efficiency. Initially, they are widely used in changing the expression of specific genes in vivo and in vitro [104]. In contrast to LVs, the nonintegrated DNA delivered by AAVs would be diluted during mitosis because of lack of integration machinery. However, it could be stably maintained in a nonintegrated form to mediate persistent gene expression in predominantly postmitotic cells [104]. With regard to damage repair and tissue regeneration, Eggers et al. used a lentiviral vector to regulate controlled expression of glial cell-line derived neurotrophic factor (GDNF), which exerts multiple effects on both Schwann cells and axons in the injured peripheral nerve [105]. Moreover, adenovirus-mediated WNT10b overexpression promoted hair follicle regeneration via the activation of the canonical WNT signaling pathway [106]. To overcome safety concerns, such as immune system activation and insertional mutagenesis, the next-generation gene therapy vectors must be developed. Hu et al. showed that a virus-biotin-avidin-biotin-material (VBABM) arrangement immobilized viral vectors on biomaterial scaffolds, that contributed to spatially control therapeutic gene delivery in bone regeneration [107]. In addition, hydrodynamic tail-vein injections of lentiviral gene delivery reduced off-target delivery and transduction in mouse liver [108].

Unlike viral vectors, non-viral vectors showed the potential to overcome many of the shortcomings of viral vectors [109]. There are a great variety of non-viral vector systems, including naked DNA or mRNA microinjection or electroporation [110], liposomes [111], cationic polymers [111], chitosan polymers [112], inorganic nanoparticles [113], transposon systems [114]. So far, the use of non-viral gene vectors has been explored in many tissue regeneration approaches, such as bone regeneration [112], cartilage regeneration [115], tendon repair [116], nerve regeneration [117]. For example, Feng et al. combined nanofibrous spongy microspheres with biodegradable nanospheres to synthesize a two-stage delivery system for plasmid DNA encoding orphan nuclear receptor 4A1 (NR4A1), and which was proved to be effective in promoting disc regeneration [118].

In recent years, exosomes have also been used as a non-viral vector of nucleic acid in regenerative medicine as an alternative to cell therapy [119]. Natural exosomes can be used to transfer small nucleic acid like siRNAs [120] and miRNAs [121], but have been shown to have issues in delivering large nucleic acids, such as plasmid DNA. Some scholars try to modify and optimize the structure of exosomes to overcome this obstacle. For example, Lin et al. developed exosome-liposome hybrid nanoparticles to deliver large nucleic acid like CRISPR-Cas9 system by increasing the binding of exosome and large nucleic acid [102].

In addition, because the messenger ribonucleic acid (mRNA) translation happens in the cytoplasm, the delivery of mRNA associated with non-viral gene delivery systems might have higher transfection efficiencies than DNA, by eliminating the need for nuclear entry. In vivo studies showed that the delivery of chemically modified ribonucleic acid (cmRNA) significantly enhances bone regeneration when compared with that of conventional plasmid DNA [122, 123].

### Transplantation of Genetically Modified Cells

Due to safety concern about dissemination of the gene vectors and their adverse side-effect in non-target sites, a direct injection of the expression vector was used less than an indirect injection of cells injection. Genetically modified cell transplantation technology has been widely applied to the research of gene therapy in vitro and in vivo for many human diseases, such as junctional epidermolysis bullosa [124], metastatic cancer [125], type 1 diabetes [126]. As mentioned above, the process of tissue regeneration is regulated spatial-temporally by a variety of cytokines. Therefore, the transplantation of genetically modified cells, which express specific cytokines, could be used to promote organ and tissue regeneration (Fig. 3E). Nascent cytokines locally synthesized may have higher activity than recombinant counterparts [127].

Mesenchymal stem cells (MSCs) have many advantages in cell transplantation, such as their easy isolation and culture, secretion of a variety of cytokines, migration and homing to damaged tissues or solid tumors. Therefore, genetically modified MSCs have been used as therapeutic cytoreagents for gene therapy [128]. For example, the upregulation of BMP2 in iPSC-MSCs can promote osteogenic differentiation and bone mineralization [129]. In another study, FGF-2 transfected BMSCs effectively promoted the repair effect of avascular necrosis of femoral head in rabbits [130]. Nevertheless, MSCs are not supposed to be immune privileged, therefore the efficacy of allogeneic MSC therapies is insure in clinical treatment [131].

Both MSCs and macrophages from monocytes can produce multiple cytokines and have been used in cell therapies [132]. Ben-Mordechai et al. showed that the favorable effects of MSC therapy in myocardial infarction (MI) were mediated by macrophages [133]. Macrophage-based therapeutic strategies have been applied in regenerative medicine for a long time [134]. Cells of the monocyte-macrophage lineage play key roles in liver regeneration and function after liver cirrhosis [135]. They might act via regulating Notch and WNT signaling pathways to specify hepatic progenitor cell fate [136]. However, only few studies focused on genetically modified monocytes or macrophages. The study of Hamm et al. indicated that genetically modified macrophages expressing low levels of prolyl hydroxylase domain protein 2 (PHD2), significantly contributed to angiogenesis through the TIE2 signaling pathway [137].

Apart from MSCs and macrophages, many other cells can also be genetically modified in regenerative medicine. In a previous review, the transplantation of transfected dental pulp progenitor/stem cells with BMP genes, by electroporation or sonoporation, into the injured pulp using an appropriate scaffold could enhance reparative dentin formation [138]. Moreover, human periodontal ligament cells (hPDLCs) transfected with LV-Ctnnb lentivirus to explore the effect of its overexpression, were further confirmed to activate the canonical WNT signaling pathway and induce cementogenic differentiation in vitro and cementum regeneration in vivo [139].

### Tooth Regeneration on the Way

Besides tooth tissue regeneration, studies on regenerating a whole tooth organ are also hot topics. Nakao and colleagues engineered the bioengineered incisor tooth germ by reconstituting single cells that were isolated from the epithelium and mesenchyme of the dental germ [140]. Ikeda and colleagues transplanted a bioengineered tooth germ into the alveolar socket of an adult mouse, and the results showed that the tooth successfully erupted and achieved occlusion [141]. Further studies have shown that the bioengineered tooth had masticatory properties and responded to harmful stimuli [141]. These studies on bioengineered tooth, suggest that total dental regeneration can be reached by achieving targeted differentiation and specific expression patterns of stem cells.

Notably, tooth regeneration can only be completed by specific epithelial and mesenchymal stem cells; while, the identity of these tooth-regenerating stem cells remains largely unknown, including their capacity to induce commonly available cells to the specific status. Future works defining the essential factors and the spatial-temporal pattern that induce the cells to the specific stage, and the development of an in vivo/in vitro control release system to deliver or release the factors in a spatial-temporal pattern, would certainly shed light on tooth regeneration (Fig. 3F).

## Outlook

Several systems that are effective in vitro or in preclinical models may fail to translate into the clinic. At present, although many scholars devote themselves to study the spatial-temporal release system of cytokines in tooth regeneration, none of these scholars have developed a system that can simulate the release behavior of cytokines during tooth development. Due to the complexity of the spatial-temporal regulatory network of tooth development, identifying key and essential factors for tooth regeneration is still being intensively explored, and if successful, would make the regeneration practical by the release of limited factors. Moreover, the in vivo safety of these systems needs more evidences before clinical translation.

In future studies, researches should be focused on: 1) Defining the essential factors for tooth regeneration; 2) Characterizing the spatial-temporal dynamics of the above identified factors; 3) Developing in vivo/in vitro control release system to deliver or release the factors in a spatial-temporal pattern, as needed by combinatorial application of biomaterials, nanobiotechnology, 3D printing and other technologies. Hopefully, a spatial-temporal control release system of cytokines will be clinically applied to human tooth regeneration in the future, which will greatly improve the quality of life, especially for the edentulous patients.
